# Chiral plasmonic nanostructures fabricated with circularly polarized light

**DOI:** 10.3762/bjnano.16.154

**Published:** 2025-12-08

**Authors:** Tian Qiao, Ming Lee Tang

**Affiliations:** 1 Department of Chemistry, University of Utah, Salt Lake City, UT 84112, United States; 2 current address: Department of Chemistry and Biochemistry, University of Nevada, Las Vegas, 4505 S. Maryland Parkway, Las Vegas, NV 89154-4003, United States

**Keywords:** circular dichroism, circularly polarized light, plasmonic nanocrystals

## Abstract

Chiral plasmonic nanostructures (cPNSs) have garnered extensive interest across disciplines due to their strong interaction with circularly polarized light (CPL). Numerous fundamental studies have demonstrated the enhancement of chiroptic effects in molecular systems and quantum emitters facilitated by chiral metal nanostructures; for example, the detection of DNA at attomolar concentrations has been achieved using cPNSs. In recent years, significant advancements have been made in the colloidal synthesis of chiral plasmonic nanostructures. A noteworthy breakthrough involves the use of CPL to fabricate cPNSs. As a traceless chiral agent, CPL holds great potential for integration with nanofabrication technologies. In this review, we will summarize the progress made in fabricating cPNSs using CPL. We will discuss the mechanisms involved in the CPL-based fabrication process and share our insights regarding the outstanding questions related to cPNSs produced by CPL. Additionally, we will outline common techniques for characterizing the chiroptic effects of cPNSs in both the far field and the near field. Last, we will review the various applications of cPNSs and highlight the most promising applications of cPNSs fabricated using CPL.

## Introduction

An object is considered chiral when it cannot be superimposed on its mirror image. Homochirality is a feature of life on Earth. For example, amino acids in living organisms are almost exclusively left-handed, while sugars are predominantly right-handed [1–2]. This creates a chiral environment in the human body, where chiral drug molecules can exhibit enantioselective chemical and pharmacological behavior [3]. One example is the drug thalidomide, which was prescribed to treat morning sickness in pregnant women. One of its enantiomers was found to have sedative effects, while the other was identified as teratogenic, leading to tragic consequences, including over 10,000 severe birth defects in children [4–8].

Promoting the separation and detection of chiral molecules is essential in the pharmaceutical industry [9]. Distinguishing between enantiomers is inherently challenging due to their identical physical properties. However, chiral molecules interact with light in a polarization-dependent manner. Historically, Arago and Biot demonstrated that cut crystals can affect the polarization of light [10–11]. Subsequently, Biot showed that organic compounds, such as turpentine oil, sugar solutions, camphor, and tartaric acid, can also rotate the polarization of linearly polarized light [12], later attributed by Fresnel to birefringence in the chiral medium [13]. In 1848, Pasteur achieved the physical separation of enantiomers for the first time. In his experiments, he crystallized the racemic sodium ammonium salt of tartaric acid, forming two distinct crystals that each rotated the polarization of light in opposite manner [14].

The interaction between chiral molecules and light is experimentally characterized by optical rotation or circular dichroism (CD). However, the inherent discrepancy in size between light and molecules typically results in weak chiroptic effects for small molecules [15–16]. High concentrations of analytes or long optical path lengths are usually required to obtain reliable experimental results. These challenges can be addressed using chiral plasmonic nanostructures (cPNSs). cPNSs enhance the intensity of the electromagnetic near field of circularly polarized light (CPL), which accelerates light absorption by molecules. Additionally, the optical chirality at the near field is greater than that of the incident CPL, increasing the polarization selectivity of the interactions between molecules and CPL [17–18].

After more than two decades of development, the synthesis of cPNSs has evolved from nanolithography and self-assembly of achiral PNSs to a more efficient seeded-growth approach in a colloidal phase. The Nam group achieved a significant breakthrough in 2018 with the large-scale synthesis of highly homogenous chiral gold nanostructures using amino acids and peptides [19]. The Tatasuma group developed the first CPL-induced growth of cPNSs in the same year [20]. CPL offers several advantages over the chiral chemical precursors used in the seeded growth approach. First, CPL is traceless. Turning off the CPL removes its influence on the material system, which can simplify the characterization of the material’s chiroptical properties. Additionally, this feature allows for the convenient application of CPL in the seeded colloidal growth of cPNSs using chiral molecules. Second, CPL holds great promise for inducing the formation of chiral nanostructures during photolithography, which could reduce the costs associated with fabricating large arrays of cPNSs for chip-based chiral sensors.

cPNSs are not only valuable for sensing chiral molecules but also show promise in promoting enantioselective chemical reactions. As this field of research is still emerging, there are many opportunities to explore. To date, the enantioselectivity induced by cPNSs has primarily been attributed to their geometrical chirality, which structurally favors interactions with chiral molecules [21–22]. However, chiral-induced spin selectivity through cPNSs can potentially promote enantioselective catalytic reactions [23]. Furthermore, the chiral near field of cPNSs can also improve the enantioselectivity of photochemical transformations. Likewise, the superchiral field of cPNSs holds great potential for amplifying the selectivity of specific photophysical processes in solid-state materials.

This review article aims to provide an overview of the advances in the fabrication of cPNSs using CPL. Section 1 will present a brief background that summarizes representative fabrication technologies for cPNSs, which employ various strategies. Section 2 will explore the mechanisms by which CPL leads to the formation of cPNSs and summarize the different cPNSs synthesized using this method. Section 3 will discuss universal metrics to characterize cPNSs. Furthermore, Section 4 will examine the roles of cPNSs in various applications. Finally, we will share insights into future directions for cPNSs fabricated using CPL.

## Review

### 1 Evolution of chiral plasmonic nanostructures

**Figure 1:**
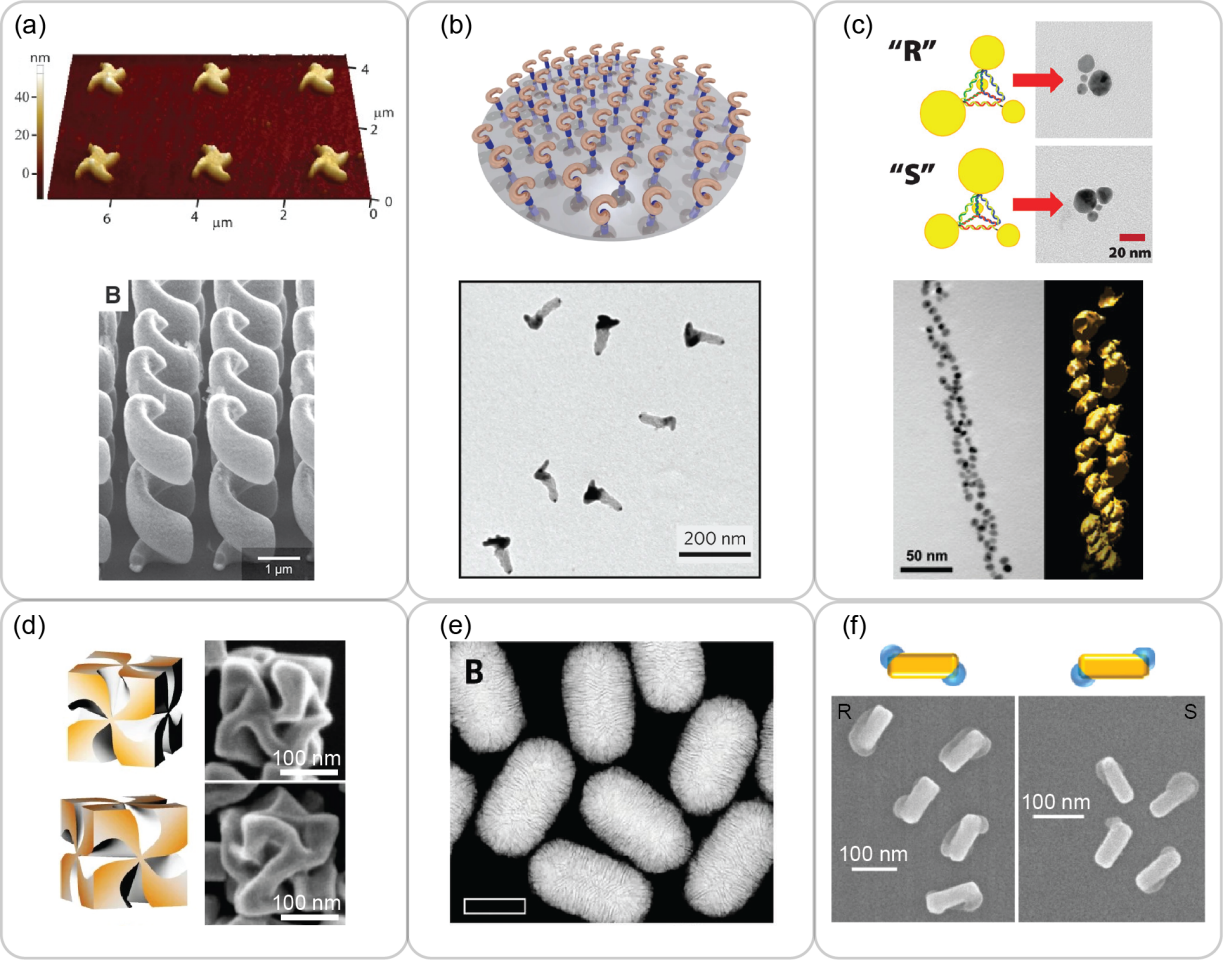
Representative cPNSs. (a) Atomic force microscopy (AFM) image of chiral gold propellers (top) and scanning electron microscopy (SEM) image of gold helices. (bottom) (b) A schematic of complex cPNSs constructed by glancing angle deposition and their transmission electron microscopy (TEM) image. (c) Top: Schematics and TEM images of pyramidal assemblies of gold NCs of different sizes. Bottom: TEM image of gold NC double helix. (d) Schematics (left) and SEM images (right) of gold helicoids obtained with glutathione. (e) Low-resolution high-angle annular dark-field scanning transmission electron microscopy (HAADF-STEM) image of chiral gold nanorods (AuNRs) obtained with cosurfactant (R)-BINAMINE. Scale bar is 100 nm. (f) Schematics (top) and SEM images (bottom) of chiral AuNR/PbO_2_ nanostructures obtained with CPL. Figure 1, panel (a) top is adapted with permission from [26] © Optical Society of America. This content is not subject to CC BY 4.0. Figure 1, panel (a) bottom was reproduced from [27] J. K. Gansel et al., *Science*, https://doi.org/10.1126%2Fscience.1177031%2C © 2009, AAAS. This content is not subject to CC BY 4.0. Figure 1, panel (b) was adapted from [28], (A. G. Mark et al., “Hybrid nanocolloids with programmed three-dimensional shape and material composition”, *Nature Materials*, Vol. 12, pages 802–807, published by Springer Nature, reproduced with permission from SNCSC). This content is not subject to CC BY 4.0. Figure 1, panel (c) top was adapted from [48], Copyright © 2009 American Chemical Society. This content is not subject to CC BY 4.0. Figure 1, panel (c) bottom was adapted from [38], Copyright © 2008 American Chemical Society. This content is not subject to CC BY 4.0. Figure 1, panel (d) was adapted from [19], (H. E. Lee et al., “Amino-acid- and peptide-directed synthesis of chiral plasmonic gold nanoparticles”, *Nature*, Vol. 556, pages 360–365, published by Springer Nature, reproduced with permission from SNCSC). This content is not subject to CC BY 4.0. Figure 1, panel (e) was reproduced from [52] G. González-Rubio et al., *Science*, https://doi.org/10.1126%2Fscience.aba0980%2C © 2020, AAAS. This content is not subject to CC BY 4.0. Figure 1, panel (f) was adapted from [20], Copyright © 2018 American Chemical Society. This content is not subject to CC BY 4.0.

Studies of chirality (both geometrical and optical) on plasmonic nanostructures date back to the 2000s. The early studies were primarily enabled by lithographically fabricated [24] and self-assembled plasmonic nanostructures [25]. Quasi-two-dimensional planar gold propellers [26] and three-dimensional gold helices [27] (Figure 1a) are two representative cPNSs fabricated by nanolithography. Using arrays of plasmonic seeds patterned by nanolithography, versatile cPNSs were also constructed with glancing angle deposition [28] (Figure 1b). Up to now, nanofabrication has produced many morphologically distinct cPNSs [29–35]. Separately, chiral organic scaffolds (e.g., DNA, DNA origami and peptides) have also been used to coax achiral plasmonic nanocrystals (NCs) into chiral assemblies [36–48]. The tetragonal and helical NC assemblies in Figure 1c are two examples. The cPNSs in Figure 1a–c and their close relatives all share a common architectural motif where their shapes imitate molecular chirality [24,49], that is, helical and tetragonal designs prevailed in these cPNSs.

A critical advance in fabricating cPNSs was the amino acid- and peptide-directed seeded growth of gold helicoids [19] (Figure 1d). This was the first time molecular chirality was transferred to plasmonic nanoparticle shapes at the single NC level. However, an atomic-level understanding of how the chiral geometry occurs is still under investigation [50–51]. Another important breakthrough is the micelle-directed seeded growth of chiral gold nanorods (AuNRs) [52]. In this approach, the surfactant of the achiral AuNR seeds, cetyltrimethylammonium chloride (CTAC), formed micelles adsorbed on NRs in the presence of dissymmetric cosurfactants that templated the growth of quasihelical shells on the NC surface (Figure 1e). These seeded-growth methods have several advantages, namely, ease of synthesis, homogeneity and large optical dissymmetry of the product, scalability, and relatively low cost. Therefore, much effort has been devoted on studying the physical properties of cPNSs, for example, ensemble CD [53], single particle level CPL scattering [54], and nonlinear chiroptic effects [55].

In 2018, the Tatsuma group reported chiral AuNR/PbO_2_ nanostructures facilitated by CPL [20] (Figure 1f). The Tang and Dionne groups built on this work to experimentally outline the importance of excitation wavelength in determining the resultant structural chirality and optical activity of chiral Au bipyramid (AuBP)/PbO_2_ nanostructures [56]. Pure metallic cPNSs constructed by CPL have also been reported [57–59]. This review’s primary focus are the fabrication details and morphology of the CPL-enabled cPNSs in Section 2.

In recent years, chiral assemblies of PNSs from mechanical forces via magnetic fields [60–61] or strain [62–63] have also been demonstrated. Although the cPNSs prepared via mechanical forces are relatively scarce, they hold great promise for achieving switchable chirality. Certain loosely related topics, such as the extrinsic chirality of PNSs [64] (chirality induced by light oblique on an achiral object), molecule-induced chirality of PNSs [65–66], and chiral metal nanoclusters [67–68], will not be discussed in detail due to the focus here on structurally intrinsic chiral plasmonic nanostructures (cPNSs).

### 2 Constructing chiral plasmonic nanostructures using CPL

Light can drive the collective oscillation of the free electrons in metals, known as surface plasmons in a plasmonic metal nanostructure. Part of the energy stored in the plasmon polaritons can be re-emitted as light, while the rest decays into electron–hole pairs. These highly energetic hot carriers can be used to drive photochemical reactions. Unlike other photocatalysts, plasmonic nanostructures are very effective in redistributing photon energy in space and localizing photochemical reactions. At the diffraction limit, light cannot be focused into a spot smaller than half its wavelength. However, waves of electrons on a metal surface can be compressed into space a tiny fraction of the wavelength of light. This allows visible and infrared light to be concentrated into regions as small as a few nanometers [69–71], enabling a significant enhancement of the electromagnetic (EM) near field. The regions of strong EM fields are termed optical hot spots of PNSs.

Under CPL illumination, the spatial distribution of hot spots adopts a chiral geometrical profile that can asymmetrically distribute photochemical reactions. Numerous numerical simulations have demonstrated the dissymmetric near-field intensity profile of PNSs under CPL. This chirality can be explained by a simple model based on classical physics, which treats transverse (T) and longitudinal (L) plasmons as classical oscillators [72–74]. The interference between these two modes defines the EM near-field intensity profile. Their interaction can be constructive or destructive depending on the phase difference between the T and L modes. CPL, equivalent to two perpendicular linearly polarized light waves with a π/2 phase difference, can further modulate the phase difference between the two modes by π/2. This generates a chiral EM field intensity profile at specific wavelengths. The full details of this model can be found in [72–74]. This model suggests that CPL spatially isolates chemical reactions by modulating the phase of plasmonic modes.

Several parameters must be carefully considered when preparing chiral PNSs with CPL. The first consideration is whether to immobilize the achiral starting PNSs on a substrate. The reactions can be easily scaled up if PNSs are dispersed in a solution. However, it is important to consider the random and dynamic orientations of NCs in the solution. The EM field distribution profile of PNSs in solution represents an average of profiles with CPL illumination from different directions, showing a compromised average geometrical dissymmetry compared to PNSs immobilized on a substrate under unidirectional CPL [75–78]. Therefore, in most cases, PNSs are immobilized on a substrate. The second parameter to consider is the energy of the excitation CPL, as it affects the EM distribution and defines the energetics of hot carriers to modulate the photochemical reaction rates. Additionally, the choice of photochemical reactions is another important consideration, as will be discussed in Sections 2.2 to 2.4.

#### 2.1 Plasmon-mediated chemical reactions

**Figure 2:**
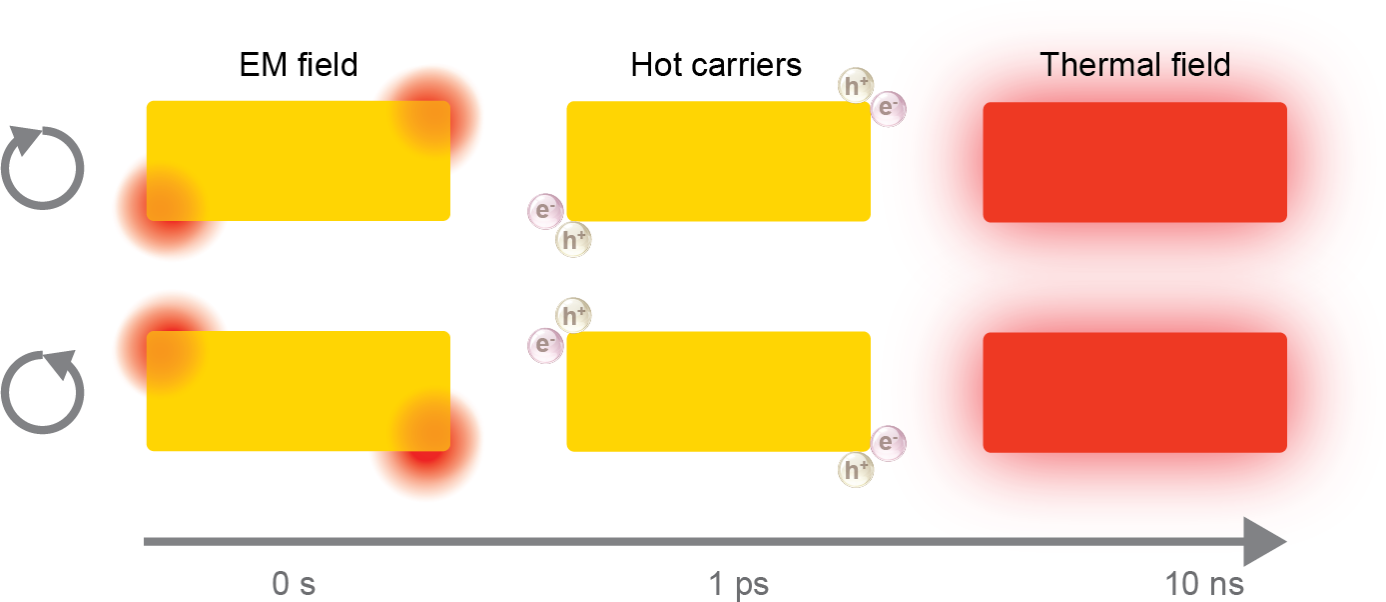
Mechanisms of plasmon-mediated chemical reactions under CPL (the polarization is arbitrary in the graph). The EM field enhancement, localized hot carrier generation, and local thermal field are depicted as the three main effects of surface plasmon excitation and relaxation, each occurring at a different time scale.

CPL creates cPNSs through plasmon-mediated chemical reactions (PMCRs). Three factors dictate photocatalysis with PNS, namely, local EM field enhancement, localized hot carrier generation, and local heating (Figure 2) [79–82]. Any of these factors can alter the landscape of a chemical reaction that might otherwise be insurmountable due to thermodynamic or kinetic barriers. The local EM field can accelerate photochemical reactions by promoting the photoexcitation of a reactant through the high photonic local density of states (LDOS). As discussed previously, CPL induces a dissymmetric EM field distribution on PNSs (Figure 2 left). This results in faster chemical conversions (several orders of magnitude) with light-absorbing reactants observed at optical hot spots compared to other locations.

Hot carriers generated in PNSs can transfer into molecular species and trigger redox reactions. The rate of hot carrier generation strongly depends on the PNS absorption, proportional to the electric field strength inside the metal. Therefore, the hot carrier generation rate profile is dictated by EM field distribution and can thus adopt a chiral geometry under CPL. However, “hot carriers” are dynamic and diffuse away from hot spots, with part of their kinetic energy lost (becoming “warm carriers”). As theoretically and experimentally demonstrated, high-energy hot carriers are better localized at the optical hot spots than warm carriers [83–87]. This means that chemical reactions that require high-energy hot carriers will be physically confined to hot spots (Figure 2 middle). This is relevant when creating chiral metal/semiconductor structures using CPL, as explained in Section 2.2. Conversely, the warm carrier distribution profile may differ from the EM field profile; this can cause photo-redox reactions to be distributed more symmetrically in space, which is not ideal for producing PNSs with large *g*-factors. Section 2.3 will address this issue with the strategy of modifying the surface ligands on PNSs to create chiral PNSs.

The non-radiative decay of hot carriers can heat the PNSs. Due to the high thermal conductivity of metals, isolated PNSs usually feature uniform temperature profiles [88–89]. Thus, the local photothermal effect at PNSs is less critical to spatially isolating chemical reactions and will not be discussed in detail. For a more comprehensive review of PMCRs generally, we refer readers to [79–82].

#### 2.2 Chiral metal/semiconductor heterostructures fabricated with CPL

**Figure 3:**
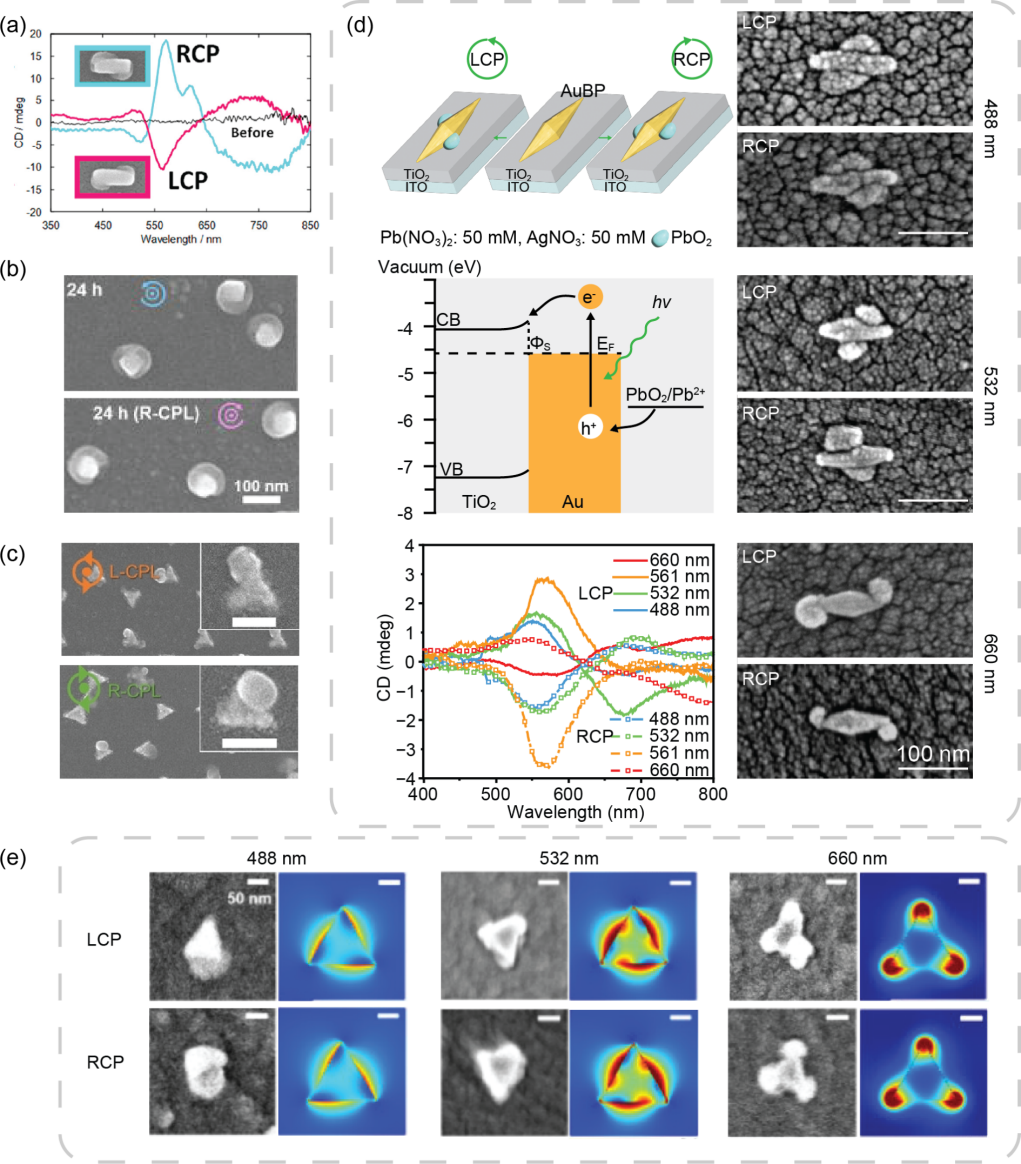
Representative chiral metal/semiconductor heterostructures fabricated by CPL. (a) SEM images and circular dichroism (CD) spectra of chiral AuNR/PbO_2_ nanostructures. (b) SEM images of chiral Au nanocube/PbO_2_ nanostructures. (c) SEM images of chiral Au triangular plate (AuTP)/PbO_2_ nanostructures. Scale bars are 100 nm. (d) Left top: schematic of the photodeposition of PbO_2_ to form chiral AuBP/PbO_2_ from achiral precursors with CPL. Left middle: an energy diagram depicting the possible mechanism of Pb^2+^ oxidation by hot holes. Left bottom: CD spectra of chiral AuBP/PbO_2_ fabricated with 488, 532, 561, and 660 nm CPL. Right panel: SEM images of chiral AuBP/PbO_2_ constructed using 488, 532, and 660 nm CPL. (e) SEM images and simulated electric field enhancement profile of Au triangle prisms using 488, 532, and 660 nm CPL. Figure 3, panel (a) was adapted from [20], Copyright © 2018 American Chemical Society. This content is not subject to CC BY 4.0. Figure 3, panel (b) was adapted from [90], with the permission of AIP Publishing. This content is not subject to CC BY 4.0. Figure 3, panel (c) was adapted from [91], T. Homma et al. “Photofabrication of Chiral Plasmonic Nanostructure Arrays”, *ChemNanoMat.*, with permission from John Wiley and Sons. Copyright © 2023 Wiley-VCH GmbH. This content is not subject to CC BY 4.0. Figure 3, panel (d) was adapted from [56], Copyright © 2024 American Chemical Society. This content is not subject to CC BY 4.0. Figure 3, panel (e) was adapted from [92], Copyright © 2025 American Chemical Society. This content is not subject to CC BY 4.0.

The Tatstuma group was the first to report cPNSs fabricated by CPL. They started with achiral AuNRs and deposited PbO_2_ at selective spots depending on the polarization of the incident light. Enantiomorphs were obtained with left and right circularly polarized light (LCPL and RCPL, respectively) (Figure 3a). In a typical experimental setup, AuNRs were immobilized on tens of nanometer thick TiO_2_ films deposited on glass. (A similar setup is shown in Figure 3d left panel for Au bipyramids (AuBPs)) Under CPL illumination, hot carriers were generated in Au. The hot electrons are diverted to the TiO_2_ electron sink, and the hot holes oxidize Pb^2+^ in solution to form PbO_2_. The TiO_2_ substrate is very efficient in removing the unwanted hot electrons to suppress carrier recombination. Ag^+^ in the reaction mixture eventually consumes these hot electrons. Previous studies have shown that the oxidation of Pb(II) to Pb(IV) is highly energy-demanding (PbO_2_/Pb^2+^: 1.46 V vs SHE); likely, it can only be initiated by hot holes, but not warm holes. As stated in Section 2.1, the photooxidation of Pb^2+^ should be orders of magnitude faster at the hot spots compared to other places. Therefore, the spatial profile of the PbO_2_ photodeposits adopts the chirality of the EM field distribution. The Tatstuma group also produced chiral Au nanocube/PbO_2_ and chiral Au nanotriangle (AuNT)/PbO_2_ heterostructures using the same strategy [90–91] (Figure 3b,c).

The chiral AuNR/PbO_2_, Au nanocube/PbO_2_, and AuNT/PbO_2_ heterostructures were fabricated using CPL from a lamp that had a broad distribution of photon energies. Despite the encouraging chiral nanostructures obtained, the mode interference model discussed at the start of Section 2 implies that broadband excitation is not ideal for fabricating cPNSs. This model describes how the magnitude and even the sign of the hot spots’ EM profile dissymmetry factor depend on the excitation wavelength. Hence, broadband CPL produces cPNSs under a combined effect of different excitation wavelengths that can sometimes compromise structural chirality and optical activity. When monochromatic excitation was used instead, distinct chiral morphologies were obtained with varying excitation wavelengths on achiral AuBPs [56] (Figure 3d, right). With blue and green CPL, PbO_2_ deposits were preferred on the lateral sides of the AuBPs. Meanwhile, with red CPL, PbO_2_ deposits were preferred on the tips of the AuBPs. These distinct chiral morphs exhibited different CD spectra. With red CPL, the chiral AuBP/PbO_2_ CD spectra were flipped in sign compared to blue and green excitation (Figure 3d left bottom panel). These results clearly outline the importance of wavelength in determining the structural chirality and optical activity of Au/PbO_2_ heterostructures. Similar wavelength-dependent chiroptic properties were obtained with chiral Au triangle prism/PbO_2_ nanostructures [92] (Figure 3e).

The CD of the chiral Au/PbO_2_ nanostructures arises from PbO_2_ modifying the dielectric constant in the vicinity of the Au nanostructures [56]. However, the drawback of the Au/PbO_2_ heterostructures is evident: PbO_2_ is an indirect-bandgap semiconductor and, therefore, does not significantly contribute to the total extinction cross section of the nanostructure compared to Ag and Au. It would be more beneficial to use pure metallic cPNSs to enhance the interaction between cPNSs and CPL.

#### 2.3 Metallic chiral nanostructures created with CPL

The light-mediated synthesis of Ag and Au NCs highlights the complexity in harnessing photoexcited hot carriers for photoredox chemistry [93–102]. When the localized surface plasmon resonance of metal seeds is excited, hot electrons reduce Ag^+^ or 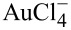 from solution and deposit metallic Ag and Au. However, various studies in the past two decades on the plasmon-mediated reduction of Ag(I) and Au(III) have demonstrated a weak dependence of the final NC morphology on the EM field distribution of symmetric nanoparticle precursors [98]. A representative example is the plasmon-mediated synthesis of Au NCs, where the surfactant polyvinylpyrrolidone (PVP) relays warm (not hot) electrons to reduce 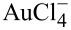 [101]. Growth was directed by the spatial distribution of PVP. Ag(I) and Au(III) reduction potentials suggest their reduction is relatively not energetically demanding. (AgCl/Ag: 0.80 V vs SHE and 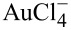/Au: 0.99 V vs SHE) [101]. As discussed in Section 2.1, these reactions can be triggered by warm electrons far from the hot spots. Indeed, [101] inferred that the sites of Au deposition (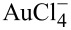 reduction) are the locations of electrons from the nanoparticle transferred via PVP. Researchers made similar observations when electron beam lithography resist was exposed to hot electrons from AuNRs [86]. This study found hydrogen silsesquioxane underwent water-assisted hydrolysis with relatively low-energy electrons (≈1.5 eV compared to >10 eV at the hot spots) at the lateral sides of AuNRs. In contrast, polymethyl(methacrylate) (PMMA) decomposed preferentially at the hot spots of the AuNRs under femtosecond laser excitation by high-energy hot electrons.

**Figure 4:**
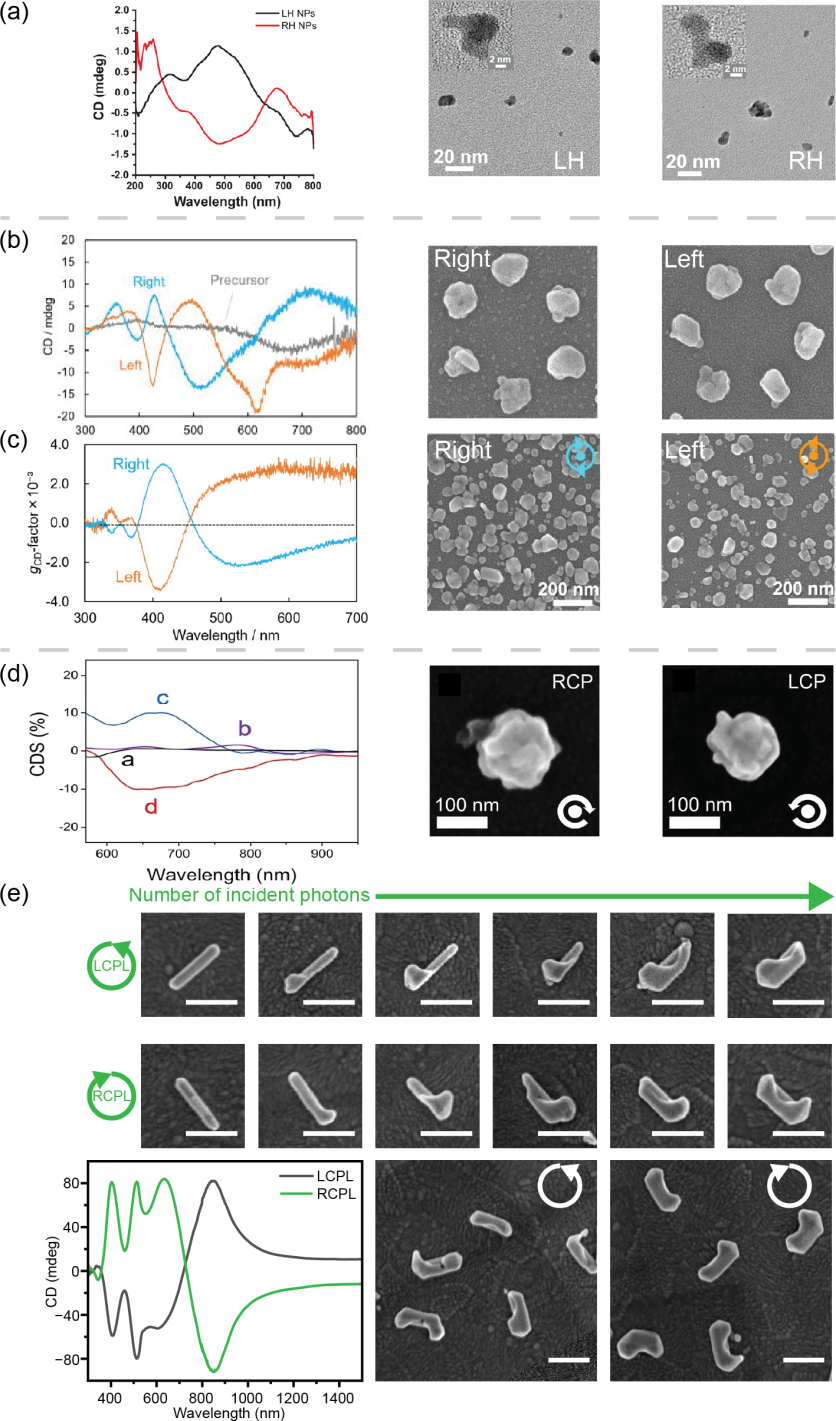
Representative metallic chiral nanostructures fabricated with CPL. (a) CD and TEM images of Au nanoparticles (NPs) produced by illuminating CPL on HAuCl_4_ solutions. (b, c) CD and SEM images of chiral Ag NPs produced on a glass slide via CPL with (b) and without (c) achiral Ag triangle plates as seeds. (d) Differential CD scattering (CDS) and SEM images of chiral Au nanocubes via illuminating CPL on achiral Au nanocubes immobilized on glass. (e) Structural evolution of chiral AgNRs fabricated with CPL and surface engineering (top two rows). CD and SEM images of chiral AgNRs after CPL illumination for 60 min (bottom). All scale bars are 200 nm in panel (e). Figure 4, panel (a) was adapted from [57], Copyright © 2019 American Chemical Society. This content is not subject to CC BY 4.0. Figure 4, panels (b) and (c) were adapted from [58], with the permission of AIP Publishing. This content is not subject to CC BY 4.0. Figure 4, panel (d) was adapted from [59], (© 2024 S. Lee et al., *Angewandte Chemie, International Edition* published by Wiley-VCH GmbH, distributed under the terms of the Creative Commons Attribution 4.0 International License, https://creativecommons.org/licenses/by/4.0). Figure 4, panel (e) was adapted from [103], (© 2025 T. Qiao et al., *Small* published by Wiley-VCH GmbH, distributed under the terms of the Creative Commons Attribution 4.0 International License, https://creativecommons.org/licenses/by/4.0).

Synthesizing chiral metallic NCs using plasmon-mediated synthesis is challenging due to reactions with nonlocalized warm electrons. Even though metallic nanostructures display optical dissymmetry in their CD spectra, electron microscopy images of the nanostructures obtained do not demonstrate clear structural handedness. The CD spectra in Figure 4a were obtained after 50 min of illuminating an aqueous solution of only HAuCl_4_ and citrate with 543 nm CPL [57]. The corresponding TEM images of the chiral AuNPs are shown in Figure 4a as the middle and right panels, described by the authors as “irregular and complex”. In the same paper, the authors attributed the formation of chiral AuNPs to the assembly of small NPs under the mechanical force of CPL. The spatial dissymmetry of hot carrier-induced chemical reactions was not discussed. While Figure 4a represents chiral metallic cPNSs synthesized in solution, Figure 4b,c show examples of chiral Ag nanostructures immobilized on a glass plate [58]. The authors fabricated these chiral nanostructures by shining CPL on glass plates immersed in an aqueous solution of silver nitrate and sodium citrate with Ag nanoplates as the precursor to obtain the arrays of chiral nanostructures in Figure 4b. No NP seeds nor organic ligands were used to obtain the chiral nanostructures in Figure 4c. The authors of the original paper commented that these nanostructures were difficult to classify into left- or right-handedness. When Au nanocubes were immobilized on ITO substrates, chiral Au nanocubes exhibiting complex shapes in SEM images were synthesized using an aqueous solution of HAuCl_4_, methanol, and PVP with CPL [59]. The optical activity of the nanocubes was observed clearly in single-particle differential circular dichroism scattering spectra, as shown in Figure 4d.

Optically chiral nanoparticles that lack clear structural chirality limit the understanding of structure–property relationships. To increase the structural chirality in metallic chiral nanostructures, we recently developed an approach to control NC surface passivation to achieve higher dissymmetry in the spatial profile for Ag photoreduction [103]. We found that the preferential stabilization of the lateral sides of Ag NRs by hexadecyltrimethylammonium bromide (CTAB) led to Ag reduction selectively on the two tips. Under CPL, this ligand passivation-mediated growth generated a “hook”-shaped nanostructure at an early stage of the photochemical reactions. This tip-preferred growth aligns with the colloidal synthesis of anisotropic Ag NCs from pentagonal seeds in the presence of CTAB/CTAC [104–105]. Growth of the hook at the tips of the AgNR nanostructures leads to a larger EM field enhancement near the tips and EM field dissymmetry on the surface of the nanostructures. This EM field distribution dominates better in the reduction of Ag downstream, resulting in a helical twist nucleating from the Ag hook that propagates along the length of the AgNR. Control experiments further strengthened our hypothesis, where relatively isotropic growth of Ag was observed under CPL without CTAB passivation. The structural evolution of the chiral AgNRs is shown in the top two rows of Figure 4e. The nanostructures obtained with CPL of opposite handedness are clear enantiomorphs of each other at each stage. Intense CD signals were collected from these chiral AgNRs. To date, these chiral AgNRs have relatively larger optical activity due to their enhanced structural chirality compared to other cPNSs fabricated with CPL. Table 1 lists the optical *g*-factors of all chiral nanostructures in Figure 4. The *g*-factor of the Au NPs in Figure 4a was not reported. The authors of [59] conducted single-particle circular differential scattering spectroscopy (CDS) on their Au nanocubes.

**Table 1:** Maximum optical *g*-factors of metallic chiral nanostructures fabricated with CPL.

Nanostructures	Maximum *g*-factor	Reference

chiral Au NPs in Figure 4a	NA	[57]
chiral Ag nanostructure arrays in Figure 4b	0.0015	[58]
chiral Ag nanostructures in Figure 4c	0.003	[58]
chiral Au nanocubes in Figure 4d	0.2 (CDS)	[59]
chiral Ag NRs in Figure 4e	0.05	[103]
chiral nanobars from a colloidal dispersion	0.0025	[77]
chiral AuNR@Ag NPs from a colloidal dispersion	0.0001	[78]

In a recent study, enhanced structural chirality of all-metal PNSs was achieved through galvanic replacement reactions (GRRs) modulated by CPL when achiral seeds were immobilized on a substrate [77]. Unlike the plasmon-mediated chemical reduction of Ag(I) or Au(III), previous studies have shown that GRRs have better spatial localization from plasmon excitation [106]. Therefore, GRRs are an effective method for synthesizing chiral metallic nanocrystals using CPL. Another recent research reduced Ag(I) on the surface of AuNRs dispersed in solution with the presence of citric acid and CPL [78]. The chiral NPs obtained in these two studies exhibit relatively low *g*-factors, measured at 0.0025 and 0.0001, due to interparticle interactions and Brownian motion of the particles diluting the effect of CPL.

The synthesis of chiral nanostructures using CPL can be scaled up by illuminating a larger area of substrates with achiral seeds. Other methods for scaling up the synthesis, such as increasing the density of achiral seeds on the substrates or conducting the synthesis in solution, are not feasible for CPL-induced chiral structures. Increasing the seed density on the substrates reduces interparticle distance, leading to the formation of achiral dimers, trimers, or even larger clusters. These clusters will have different hot carrier distribution profiles, resulting in varied shapes of the chiral structures. Consequently, the shape consistency of CPL-induced chiral structures will be compromised. The degree of symmetry breaking in CPL-induced chiral structures is lower when dispersing achiral seeds in solution compared to depositing them on a substrate [77].

The CD spectra of nanostructures in Sections 2.2 and 2.3 were collected on their thin films deposited on the substrate, with the exceptions of the AuNP in Figure 4a, chiral nanobars, and chiral AuNR@Ag NPs in Table 1. The authors of [28] provided an approach that can potentially redissolve CPL-induced chiral nanostructures in solutions by sonicating the substrates in an aqueous solution of surfactant.

#### 2.4 Plasmon-mediated polymer motions and polymerization using CPL

**Figure 5:**
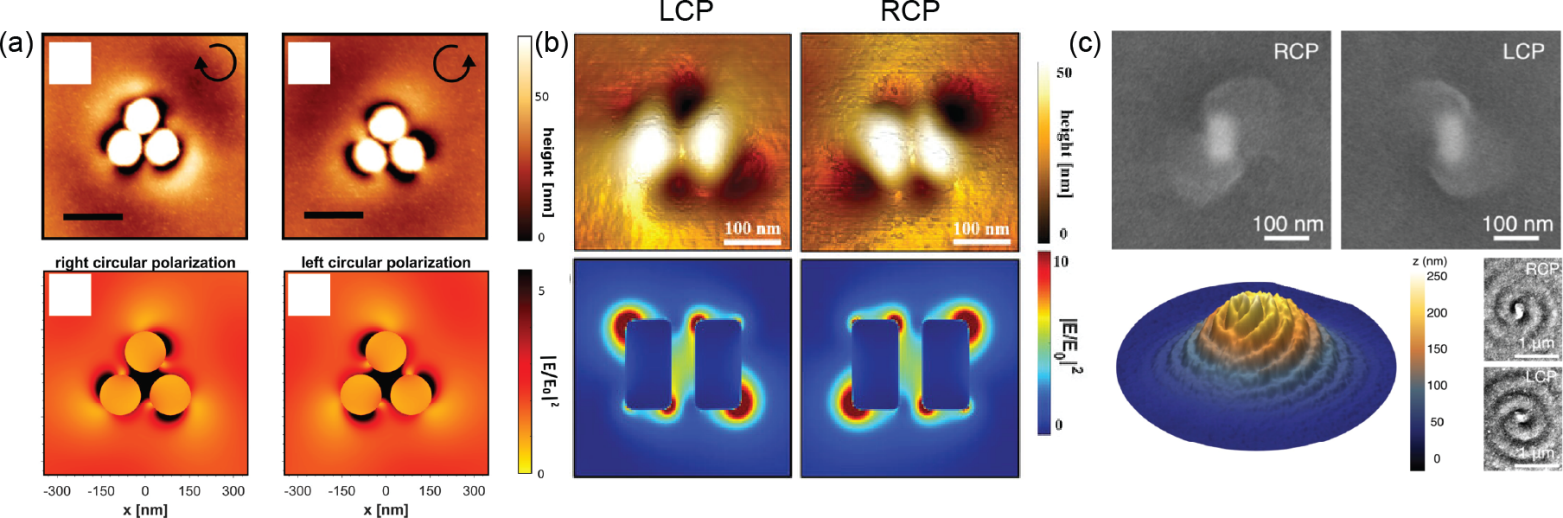
(a, b) Top: Atomic force microscopy (AFM) images of Au metamolecules with photosensitive azobenzene-containing polymer displaced under CPL. Bottom: electric field enhancement simulation of Au metamolecules under CPL. (c) SEM and AFM images showing the spiral polydivinylbenzene with the achiral Au@Ag nanoparticle in the middle after CPL illumination. Figure 5, panel (a) was adapted from [107], Copyright © 2020 American Chemical Society. This content is not subject to CC BY 4.0. Figure 5, panel (b) was adapted from [108], T. Aoudjit et al. “Photochemical Imaging of Near-Field and Dissymmetry Factor in Chiral Nanostructures”, *Advanced Optical Materials*, with permission from John Wiley and Sons. Copyright © 2023 Wiley-VCH GmbH. This content is not subject to CC BY 4.0. Figure 5, panel (c) was adapted from [109], (© 2024 H.-Y. Ahn et al., Published by American Chemical Society, distributed under the terms of the Creative Commons Attribution-NonCommercial-NoDerivatives 4.0 International License, https://creativecommons.org/licenses/by-nc-nd/4.0/). This content is not subject to CC BY 4.0.

The enhanced EM field under CPL of achiral PNSs can result in a spatially dissymmetric distribution of light absorption by chromophores. As depicted in Figure 5a, CPL resulted in a chiral electric field distribution around an achiral gold meta molecule comprised of three gold discs [107]. This meta molecule was coated with an azobenzene-containing polymer that undergoes a conformational change upon photon absorption, inducing molecular movement. This movement can lead to a significant displacement of the polymer backbone, with the degree of displacement being dependent on the strength of the electric field under CPL. Chiral topographic changes due to the polymer displacement were observed using atomic force microscopy (AFM). Similar observations were made on two coupled AuNRs, as illustrated in Figure 5b [108]. On a larger spatial scale (micrometers), the interaction between the incident CPL and the CPL scattered by the achiral Au@Ag nanoparticle resulted in a double spiral pattern of the polymer coating (Figure 5c) [109]. The photoinitiator tetraphenylporphyrin absorbs light and reacts with the monomer divinylbenzene at its excited state, leading to polymerization. The photopolymerization yield is proportional to the EM field, demonstrating a spiral profile after CPL illumination.

The authors aimed to use plasmon-mediated polymer motion, as demonstrated in Figure 5a,b, to visualize the near field of PNSs. We discussed these two studies alongside other fabrication methods because they involved plasmon-driven processes. In addition to creating cPNSs, CPL was also found to effectively modify or enhance the structural chirality and optical activity of cPNSs synthesized with chiral molecules present [110–111]. This understanding about how CPL dictates chiral structure formation is also applicable to nanofabrication technologies. For example, the chiral EM near field or photoelectron profile can create chiral patterns in photolithography and electron-beam lithography in the proximity of PNSs.

### 3. Theoretical and experimental characterization of chiral plasmonic nanostructures

Characterizing cPNSs is crucial for informing the synthesis and uses of cPNSs. This section on different characterization techniques will not be restricted to cPNSs created with CPL, as the techniques below can be universally employed to investigate the chirality-related properties of any PNSs.

#### 3.1 Theoretical simulation of cPNSs

Simulation methods have proven to be very useful for interpreting the intricacies of photon–matter interactions in PNSs and describing their optical properties [112–113]. The absorption and scattering of a spherical particle are described using Mie theory [114], which solves Maxwell’s equations to predict how light interacts with the particle with a multipole expansion of the EM fields. For ellipsoids that are much smaller than the wavelength of light and experience a uniform induced EM field (in the quasistatic regime), the Gans model provides an analytical solution for their absorption and scattering. When dealing with more complex shapes, numerical approaches such as the discrete dipole approximation, the finite difference time domain method, the finite element method, and the boundary element method are used to describe the optical properties of PNS. Numerous comprehensive review articles exist to compare different simulation methods, so we will not delve into detail here.

**Figure 6:**
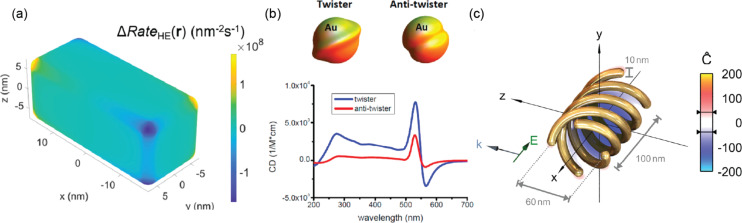
(a) Map of the differential hot electron excitation rate (Δ*Rate*_HE_(r)) between LCPL and RCPL incidence for a rectangular Au prism. (b) Simulated CD spectra of a gold twister and an anti-twister. (c) 3D map of the normalized optical chirality around a four-helix structure on resonance with a pitch of 100 nm. Figure 6, panel (a) was adapted from [75], (© 2021 L. V. Besteiro et al., Published by American Chemical Society, distributed under the terms of the Creative Commons Attribution 4.0 International License, https://creativecommons.org/licenses/by/4.0). Figure 6, panel (b) was adapted from [115], Copyright © 2012 American Chemical Society. This content is not subject to CC BY 4.0. Figure 6, panel (c) was adapted from [125], Copyright © 2014 American Chemical Society. This content is not subject to CC BY 4.0.

To understand how structural chirality forms using CPL, simulations were carried out to predict the physical locations of PMCRs through the distribution of the electric field and hot carriers. In Section 2, we discussed the physical insights of the chiral EM field distribution of achiral PNSs under CPL, based on a plasmon interference model derived from classical physics. The different shapes, sizes, and dielectric environments of the achiral PNSs were more accurately accounted for using numerical simulations. Govorov et al. described the inhomogeneous distribution of hot electrons in PNSs under CPL [75–76]. The map of the differential hot electron excitation rate on an Au rectangular prism is shown in Figure 6a. The rate of hot electron excitation was calculated considering the Fermi level of the metal, the energy difference between the acceptor state and the Fermi level, the energy of the incident light, and the local electric field strength. It was shown that the shape of the metal seeds and the experimental conditions, such as depositing the seeds on a substrate or dispersing them in a solution, affect the distribution of hot carriers and the dissymmetry of the resulting chiral cPNSs.

Simulations have also been conducted to understand the optical properties (e.g., CD and *g*-factor) of existing cPNSs and to make predictions for cPNSs with large optical activity. Even before any plasmonic nanocrystals with intrinsically chiral shapes were synthesized, Govorov et al. predicted the CD spectra of Au twisters with numerical simulations (Figure 6b) [115]. The simulations considered the polarization vectors of the incident light. The dissymmetric shapes of the gold nano-twisters led to the mixing of different plasmon modes, generating a nonzero CD. The first step in calculating the CD of experimentally obtained cPNSs is to import the morphological features of the cPNSs into the simulation software. The morphological features are typically obtained from TEM or SEM. Ideally, electron tomography can generate accurate 3D morphological information of the cPNSs to feed into the simulation, although such analysis on nanocrystals is still far from routine. Additionally, the size and shape inhomogeneity of cPNSs will cause a discrepancy between the experimental CD data and the simulated data. Therefore, the synthesis of highly uniform cPNSs is always desirable.

Simulating and characterizing the superchiral EM field about a cPNS is of great interest. EM fields with asymmetry exceeding that of CPL are referred to as superchiral fields. The optical chirality (*C*) of a superchiral field is described in Equation 1 [116–118].


[1] $$C\equiv \frac{\varepsilon_{0}}{2}E\cdot \nabla \times E+\frac{1}{2\mu_{0}}B\cdot \nabla \times B$$

Here, ε_0_ and μ_0_ are, respectively, the permittivity and permeability of free space, and **E** and **B** are the time-dependent electric and magnetic fields, respectively. Derived by Tang and Cohen [119], the optical chirality is relevant to *g*_λ_, the fractional difference in rates of excitation of a small molecule between LCPL and RCPL at wavelength λ through Equation 2,


[2] $$g_{\lambda }=g_{\text{CPL}}\left(\frac{cC}{2U_{\text{e}}\omega }\right),$$

where *g*_CPL_ is the material dissymmetry factor, *c* is the speed of light, *U*_e_ is the local electric energy density, and ω is the angular frequency. Equation 2 shows that superchiral fields can better distinguish different enantiomers, making them useful for enantioselective sensing and chemical reactions [120–122]. Near a cPNS, usually a dominant handedness *C* is obtained with CPL chirality matching the structural chirality. By adjusting the morphological parameters of cPNSs, the net *C* in their vicinity can be optimized to enhance the overall interactions with chiral molecules (Figure 6c) [123–125].

#### 3.2 Chiroptic effects of cPNSs

**Figure 7:**
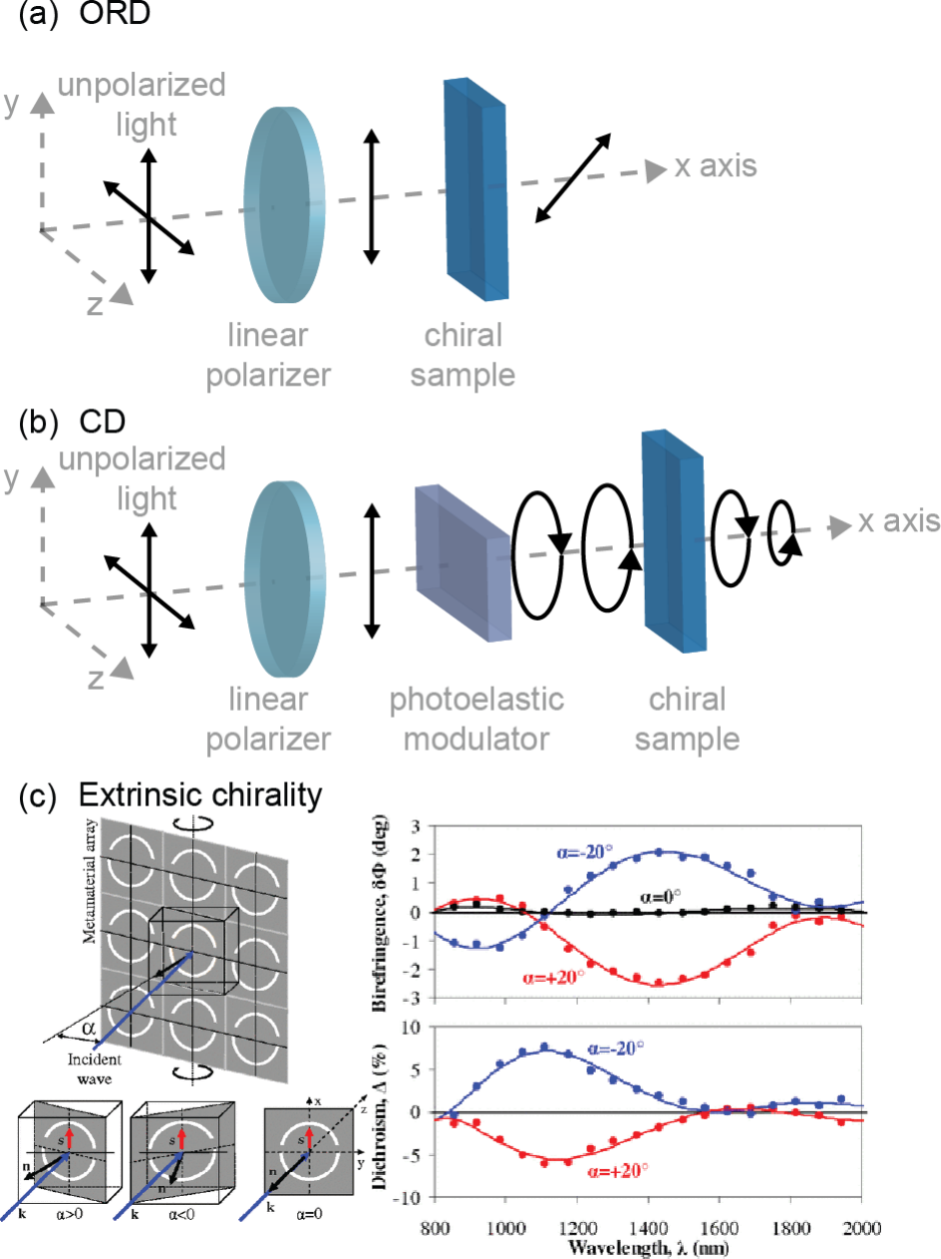
Schematics of the (a) ORD and (b) CD measurement principles. (c) An array of split rings manifests optical activity at the oblique incidence of light. Optical activity is detected when the plane of the array is tilted around the *x*-axis. Figure 7, panel (c) was adapted with permission from [64], Copyright © 2009 by the American Physical Society. This content is not subject to CC BY 4.0.

Chiral objects respond selectively to the handedness of CPL. Materials are optically active when they exhibit CD or optical rotation dispersion (ORD). The schematics of ORD and CD measurement principles are demonstrated in Figure 7a,b. Optical rotation, also known as circular birefringence (CB), refers to the rotation of the orientation of the polarization plane of linearly polarized light when traveling through a chiral medium. It is related to the real part of the material refractive index, and a chiral material has a different index for LCPL and RCPL. CB is measured through ORD expressed by Equation 3:


[3] $$\theta =\frac{\pi }{\lambda }\left(n_{\text{RCPL}}-n_{\text{LCPL}}\right)l,$$

where *n*_RCPL_ and *n*_LCPL_ are the real parts of the refractive index for RCPL and LCPL, respectively. From Equation 3, we can see that CB is also dependent on the wavelength (λ)of and the length traveled (*l*) by the EM wave.

CD is another manifestation of optical activity. It is generally defined as the differential absorption of LCPL and RCPL as expressed in Equation 4:


[4] $$\text{CD}=A_{\text{RCPL}}-A_{\text{LCPL}}.$$

For a better comparison of different optically active materials, Kuhn’s dissymmetry factor, or *g*-factor, is often used to normalize CD relative to the material’s total absorbance:


[5] $$g=2\frac{A_{\text{RCPL}}-A_{\text{LCPL}}}{A_{\text{RCPL}}+A_{\text{LCPL}}}.$$

For large cPNSs, however, scattering cannot be neglected, and a CD spectrometer often measures the differential extinction of RCPL and LCPL. Note that absorption is related to the imaginary part of the medium’s refractive index; scattering is related to the real part of the refractive index. In general, a chiral medium should express both CB and CD, two parameters are correlated by the Kramers–Kronig relation [122].

When experimentally characterizing the optical activity of cPNSs, it is critical to distinguish between intrinsic and extrinsic chirality. Extrinsic chirality refers to the chirality produced by the oblique light incident on the surface normal of the material studied, while the material is an achiral, periodically ordered surface (Figure 7c) [64]. Its strength depends on the angle of light incidence. Ignoring extrinsic chirality may lead to the misinterpretation of CD or ORD spectra. It is also essential to consider morphological inhomogeneity when analyzing the optical activity of cPNSs. Single-particle CDS has shown that a chiral Au helicoid can have up to four times larger *g*-factor of chiral light scattering obtained compared to its ensemble [54].

#### 3.3 Characterizing the near field of cPNSs

Section 3.2 discussed the chiroptic effects of cPNSs in the far field. This section will cover the experimental methods for observing the near fields of cPNSs. Advanced techniques, such as near-field scanning optical microscopy (NSOM), cathodoluminescence (CL), electron energy loss spectroscopy (EELS), and photoemission electron microscopy (PEEM), offer spatial resolution of 10 nm or better to characterize plasmon near fields. These techniques can be readily applied to cPNSs to map the chirality of the near-field geometry or the near-field interaction with CPL.

**Figure 8:**
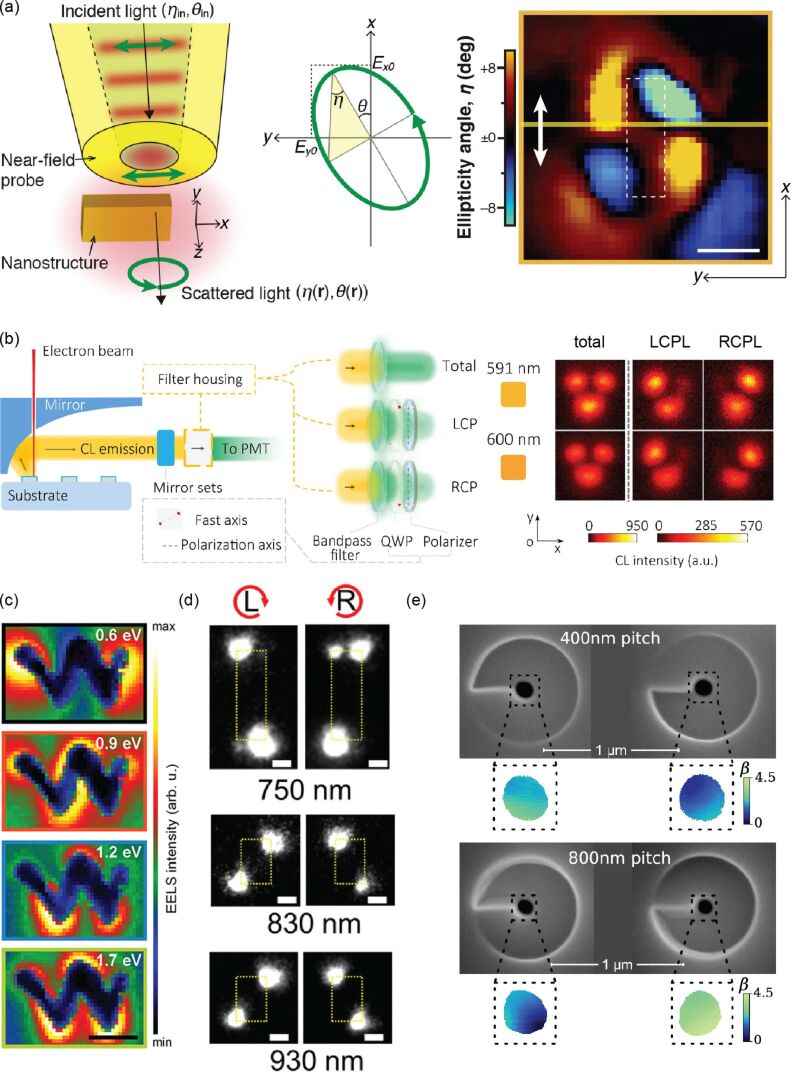
(a) Experimental scheme of near field polarimetry measurements using NSOM and the ellipticity angle image for a single gold nanorectangle irradiated with linearly polarized light (white arrow). (b) Experimental scheme of CPL detection using CL in an SEM and the circularly polarized CL intensity map on an achiral V-shaped Al nanoantenna at different detection wavelengths. (c) Energy-filtered EELS intensity images on a left-handed Au nanohelix. (d) Integrated PE signals around Au NRs under CPL at various wavelengths. (e) SEM images of Au spirals with varying pitch sizes and their PINEM maps under LCPL. Figure 8, panel (a) was adapted from [126], Copyright © 2018 American Chemical Society. This content is not subject to CC BY 4.0. Figure 8, panel (b) was adapted from [129], Copyright © 2018 American Chemical Society. This content is not subject to CC BY 4.0. Figure 8, panel (c) was adapted from [132], (© 2023 R. Lingstädt et al., Published by American Chemical Society, distributed under the terms of the Creative Commons Attribution 4.0 International License, https://creativecommons.org/licenses/by/4.0). Figure 8, panel (d) was adapted from [72], (© 2021 T. Oshikiri et al., Published by American Chemical Society, distributed under the terms of the Creative Commons Attribution-NonCommercial-NoDerivatives 4.0 International License, https://creativecommons.org/licenses/by-nc-nd/4.0/). This content is not subject to CC BY 4.0. Figure 8, panel (e) was adapted from [135], T. R. Harvey et al., “Probing Chirality with Inelastic Electron-Light Scattering”, *Nano Letters*, Copyright © 2020 American Chemical Society, distributed under the ACS AuthorChoice/Editors’ Choice via Creative Commons Attribution Non-Commercial No Derivative Works 4.0 Usage Agreement, https://pubs.acs.org/page/policy/authorchoice_ccbyncnd_termsofuse.html. This content is not subject to CC BY 4.0.

NSOM is a scanning probe technique that overcomes the diffraction limit in traditional far-field optical microscopy by exciting samples with an evanescent field. An optical image with tens of nanometers or higher spatial resolution is generated with a probe scanning the sample, recording the light-matter interaction in the near field of the sample. Polarimetry of the optical near field can be collected using NSOM. Figure 8a maps the near-field ellipticity about an achiral gold nanorectangle under linearly polarized light excitation [126] and illustrates the experimental principle with the definition of the ellipticity angle. The polarimetry map of the NSOM image is qualitatively in line with previous theoretical studies that predict the superchiral field of an achiral metal nanostructure under linear polarized excitation [123–124]. On cPNSs, the polarimetry map can also be obtained with NSOM using incident light with different polarizations.

CL images of a PNS portray its wavelength-dependent photonic LDOS [127]. The spatially localized electron beam excitation on PNSs allows for nanometer-scale spatial resolution. When an electron beam is incident on PNSs, plasmon modes are excited, and radiative photon emission is detected [128]. The intensity of this emission depends on LDOS. The polarization of CL can be resolved to correlate the chirality of the local electric field distribution to CPL excitation on PNSs [129–130]. The correlation between the chiral LDOS distribution and polarization-resolved CL is based on the reciprocity relation between electron beam excitation and optical plane wave illumination. Figure 8b shows an example of the circular polarization-resolved CL map on achiral Al V-shaped nanoantennas. Guided by the reciprocity theorem, the chiral CL spatial distribution can also be explained by the interference of plasmon modes.

NSOM and CL collect photons in the near field of PNSs, whereas EELS and PEEM collect electrons. EELS also provides insight into the energy-dependent LDOS [131]. The energy-filtered EELS probability map of an Au nanohelix is shown in Figure 8c [132]. PEEM analyzes the spatial distribution of electrons ejected by samples under light excitation [133]. Under CPL, the map of the electron emission intensity from achiral AuNRs adopts a chiral geometry, as shown in Figure 8d [72]. PEEM can produce time-resolved data to probe the dynamics of photoemission [134]. In addition to EELS and PEEM, the so-called photon-induced near-field electron microscopy (PINEM) measures the interaction between electrons and cPNSs upon laser excitation by mapping the EELS within an electron microscope [135] (Figure 8e).

In summary, we have reviewed the nanoscale characterization tools correlating structure to chiroptical activity of PNSs in this section. CD and ORD are handy tools for describing the optical activity of PNSs, determined by their geometrical chirality, whether intrinsic or extrinsic. Characterizing the near-field spatial distribution dissymmetry and near-field polarization with NSOM, CL, EELS, PEEM, and PINEM provides valuable insight on the interaction of molecular species or quantum emitters with PNS. The experimental results and theoretical simulations will guide the fabrication of cPNSs with the optimal enantioselectivity.

### 4 Applications of chiral plasmonic nanostructures

In line with our discussions in previous sections, we will summarize the representative applications of cPNSs based on the related types of material properties. We refer the readers to the comprehensive reviews [18,120–122] for applications of cPNSs categorized by interest.

#### 4.1 Applications based on the geometrical chirality of cPNSs

Several recent studies have shown that the geometrical chirality of cPNSs leads to enantioselective interaction with molecules. For example, Xu et al. have shown that Au nanoparticles of opposite handedness differ in their ability to activate immune systems [110]. The authors suggested the left-handed nanoparticles had stronger binding affinities to two proteins due to the supramolecular interactions between the chiral extracellular domains and the curved chiral nanoparticles, which led to the enantiomer-dependent immunological response. Nam et al. have shown that the stronger binding affinities of molecules to regions of both positive and negative surface electrostatic potential in the vicinity of kink atoms on the surface of Au helicoids led to higher catalytic activity for the oxidation of glucose [22]. Moreover, Au helicoids synthesized with ʟ-glutathione exhibited higher catalytic activity for ʟ-glucose than for ᴅ-glucose. Huang et al. have demonstrated the enantioselective photocyclodimerization of 2-anthracenecarboxylic acid adsorbed on Ag nanohelixes [21]. The authors attributed the enantioselectivity to the different adsorption energies of stacked dimer precursors arising from the different geometrical adhering to the wave-like chiral lattices on the nanohelix surface. The authors also suggested that the superchiral near fields may contribute to this study.

Applications discussed in this section mainly focus on the local chiral geometric domain rather than the overall geometrical chirality of the nanostructure. This is likely because of the size mismatch between most plasmonic nanostructures and chiral molecules, making the direct link between nanostructural chirality and molecular chirality challenging. Additionally, the overall geometrical chirality of a plasmonic nanocrystal can be characterized by electron microscopy and optical spectroscopy, such as CD. However, characterizing local chiral domains can be more challenging. References [21–22] have offered valuable insights through simulations, crystallographic analysis, and high-resolution TEM. A more general model would be desirable through further in-depth studies.

#### 4.2 Applications based on the circular differential extinction of cPNSs

**Figure 9:**
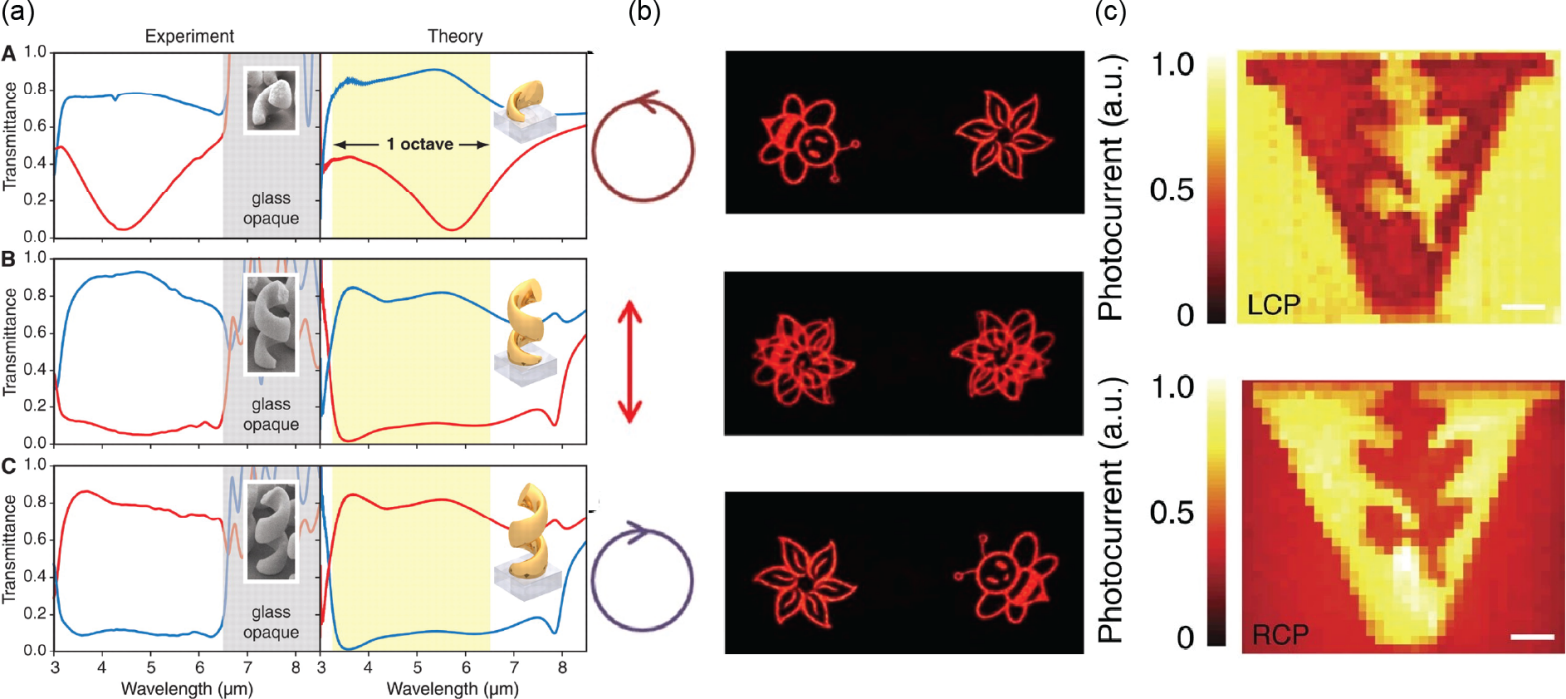
(a) Experimental and calculated transmittance spectra of LCPL (red) and RCPL (blue) measured with normal incidence on left-handed less-than-one-pitch helices (top), two pitches of left-handed helices (middle), and two pitches of right-handed helices (bottom). (b) Different images were reconstructed through a chiral metasurface depending on the polarization of the incident light. (c) Photocurrent maps of a metamaterial under CPL. Scale bars are 10 μm. Figure 9, panel (a) was reproduced from [27] J. K. Gansel et al., *Science*, https://doi.org/10.1126%2Fscience.1177031%2C © 2009, AAAS. This content is not subject to CC BY 4.0. Figure 9, panel (b) was adapted from [138], (© 2015 D. Wen et al., published by Springer Nature, distributed under the terms of the Creative Commons Attribution 4.0 International License, https://creativecommons.org/licenses/by/4.0). Figure 9, panel (c) was adapted from [141], (© 2015 W. Li et al., published by Springer Nature, distributed under the terms of the Creative Commons Attribution 4.0 International License, https://creativecommons.org/licenses/by/4.0).

The strong interaction between cPNSs and CPL makes them ideal for polarization optics [27,136–137]. Figure 9a shows an example of a broadband circular polarizer made with arrays of Au helices. CPL illuminating the metamaterial with the same handedness as the helices is preferentially absorbed and scattered, whereas that with the opposite handedness is preferentially transmitted. The circular polarization-dependent extinction of cPNSs has also been used for multiplexing holograms [138–140]. As shown in Figure 9b, a metasurface composed of AgNRs allows for the reconstruction of two sets of hologram patterns by switching the circular polarization of incident light. The large circular differential absorption of cPNSs also leads to a polarization-dependent differential hot carrier generation rate that can be used to build CPL photodetectors [141–142]. Figure 9c demonstrates an example. Depending on the chirality of the enantiomers comprising the pattern, photocurrents of different intensities were generated under CPL. Similarly, the circular polarization-sensitive photothermal effect has potential for photobolometers [143–145].

Ohmic losses and the high fabrication cost of large-scale cPNSs pose potential challenges for their use in polarizers or photodetectors. CPL-induced cPNSs are promising for reducing the fabrication costs of polarization optics based on plasmonic nanostructures. The ohmic loss can be partly mitigated by improving the crystallinity of cPNSs through optimizing synthesis parameters.

#### 4.3 Applications related to the superchiral near field of cPNSs

Metamaterials made of cPNSs have been extensively studied for their ability to detect chiral molecular species [53,146–152]. In Section 3.1, it is discussed that the optical chirality of the superchiral field is larger than that of the incident CPL, leading to an increase in the dissymmetry in the excitation rate of molecules. The near-field optical chirality is typically 10 to 100 times greater than that of the incident CPL, but this alone cannot explain the over six orders of magnitude enhancement [53] in sensitivity observed when detecting chiral molecules. Research has shown that the Coulomb interaction between PNSs and molecules induces CD on achiral PNSs or achiral molecules when the other component is chiral [18,153–155]. In addition, when molecules are attached near the cPNSs, their CD spectra shift due to modifications in the dielectric constant. All these factors contribute to the spectral changes observed when cPNSs detect chiral molecules. Still, it is important to point out that cPNSs are very useful for generating detailed information on biomolecules, such as the hierarchy of protein structures [146–148].

The superchiral field of cPNSs has also been utilized to selectively modulate the excitation and spontaneous emission rate of valley excitons in transition metal dichalcogenide (TMDC) monolayers [156–160]. TMDCs have two inequivalent valleys in the Brillouin zone, each selectively coupling with one CPL. The extent of valley polarization under CPL excitation and valley-polarized photoluminescence (PL) in TMDCs can be increased by coupling to cPNSs. It has been suggested that cPNSs contribute to the enhancement of the degree of circular polarization by suppressing the intervalley scattering rate and accelerating the valley-polarized exciton decay rate. Though the circular polarization-selective PL of achiral quantum dots has been demonstrated when coupled to cPNSs [161–164], the underlying mechanism still needs to be explored.

## Conclusion – Remarks and Perspectives

The field of research on chiral plasmonic nanostructures (cPNSs) is rapidly expanding. The colloidal synthesis of cPNSs using chiral molecules has reached a stage where new shapes are being developed to target specific functions. Ongoing studies are aimed at understanding the atomic-level formation of chiral structures. There is still significant potential for future progress using CPL to construct cPNSs. For example, the *g*-factor of cPNSs still has room for improvement by adjusting the incident light and the chemical conditions. Additionally, there is great potential for using CPL in nanofabrication technologies to produce cPNSs. For cPNSs, detecting their far-field optical activity helps guide material synthesis, and their near-field optical chirality is relevant for sensing chiral molecules. Another important application of cPNSs is the enhancement of CPL emission from quantum emitters. In this way, not only is the selectivity of the circularly polarized emission enhanced, but the spontaneous emission of quantum emitters is accelerated due to the Purcell effect.

Finally, we would like to address issues at the forefront of CPL-generated cPNSs in the coming years: (i) Establishing experimental parameters essential to maximizing the *g*-factor of cPNSs synthesized using CPL. To date, we have demonstrated the impact of excitation wavelength and surface passivation on the morphologies of cPNSs and their resulting *g*-factors. However, gaps in knowledge still need to be addressed to provide complete guidance for synthesizing cPNSs approaching the theoretical maximum *g*-factor of 2. For instance, the geometry of the initial achiral PNSs can be optimized to achieve greater dissymmetry in the electric field distribution when exposed to CPL. Additionally, the local thermal effects that may lead to isotropic photochemical deposition on the achiral PNSs require further investigation and must be taken into account. Furthermore, the interactions between the achiral PNS seeds and the substrates on which they are immobilized should be carefully explored to enhance charge separation and achieve better distribution dissymmetry of hot carriers. These insights will also be valuable for integrating CPL with chiral molecular templates for growing cPNSs. (ii) Periodic arrays of cPNSs can be fabricated using CPL on a substrate. Achiral seeds can be patterned using nanoimprint lithography or assembling colloidally synthesized nanoparticles into lithographically defined templates [165–167]. The cPNSs can then be constructed under CPL illumination in a solution phase. Compared to purely lithographical methods, this fabrication method is cost-efficient and less time-consuming. It will also produce periodic arrays of cPNSs with higher crystallinity and finer control over their morphologies. (iii) CPL-fabricated cPNSs are highly suitable for using light to switch chirality. In 2020, the Tatsuma group demonstrated a chiral system of AuNR and PbO_2_ that exhibited switchable chirality [168]. In their research, the PbO_2_ in left-handed AuNR/PbO_2_ heterostructures were dissolved through reductive reactions when exposed to UV illumination. Subsequently, the right-handed heterostructure could be fabricated using RCPL. Similar concepts can be utilized to fabricate switchable all-metallic chiral nanostructures. The two structural handedness can serve as “on” and “off” states for data storage applications. (iv) Chiral plasmonic metamaterials are promising platforms for on-chip biomolecular sensing applications. A primary challenge in detecting chiral molecules is the limited ability to directly measure the enhanced CD spectra of the analyte. Biomolecule detection primarily relies on changes in the line shape of the metamaterial spectra. This provides limited information about molecular structure [18]. Therefore, additional fundamental studies are needed to develop material platforms to advance more complicated commercial applications. Integrating nanolithography with CPL-induced solution synthesis can facilitate the construction of chiral metamaterials. This approach will accelerate material development and enhance the potential for real-world applications by reducing fabrication costs and allowing for greater flexibility in tuning material geometries through controlled illumination parameters. (v) Plasmonic catalysis has emerged as an exciting new field within heterogeneous catalysis, offering significant advantages for precision chemistry. Plasmonic catalysis harnesses energetic charge carriers, photons, and phonons generated by light excitation, potentially allowing for a level of control greater than what can be achieved with purely thermally driven catalysis [169]. cPNSs are promising catalysts for enantioselective chemical reactions. In addition to their geometrical selectivity, cPNSs can enhance the enantioselectivity of chemical reactions through two other mechanisms, that is, the superchiral field, which increases molecular interaction selectivity with CPL [117,119], and the production of spin-polarized hot carriers due to chirality-induced spin selectivity [23]. As a new and exciting research direction, many fundamental questions still need to be addressed. Chiral plasmonic metamaterials created using CPL will serve as valuable platforms to experimentally explore the potential of cPNSs for enantioselective catalytic reactions.

## Data Availability

Data sharing is not applicable as no new data was generated or analyzed in this study.
